# SMILE: neural signal acquisition and intra-body transmission for facial nerve bypass—An acute feasibility study and proof-of-concept in a rat model

**DOI:** 10.3389/fnins.2026.1798238

**Published:** 2026-06-30

**Authors:** Giorgia Faravelli, Francesca Talpo, Anna Marcucci, Mauro Marchese, Morgan Scollo, Giovanni Magenes, Laura Galluccio, Federico Biglioli, Fabiana Allevi, Gerardo Rosario Biella, Maurizio Magarini, Pietro Savazzi, Anna Vizziello, Paolo Spaiardi

**Affiliations:** 1 Department of Biology and Biotechnology Lazzaro Spallanzani, University of Pavia, Pavia, Italy; 2 Department of Electrical, Computer and Biomedical Engineering, University of Pavia, Pavia, Italy; 3 Department of Electrical electronic and computer engineering (DIEEI), National Inter-University Consortium for Telecommunications (CNIT), University of Catania, Catania, Italy; 4 Department of Maxillo Facial Surgery, ASST Santi Paolo e Carlo, University of Milan, Milan, Italy; 5 Istituto Nazionale di Fisica Nucleare, Sezione di Pavia, Pavia, Italy; 6 Department of Electronics, Information and Bioengineering, National Inter-University Consortium for Telecommunications (CNIT), Polytechnic University of Milan, Milan, Italy; 7 Fondazione I.R.C.C.S. Policlinico, San Matteo, Pavia, Italy; 8 Department of Electrical, Computer and Biomedical Engineering, National Inter-University Consortium for Telecommunications CNIT, University of Parma, Parma, Italy

**Keywords:** facial palsy, galvanic coupling, implantable devices, intra-body communications, neural stimulation, ultrasounds, vibrissal protocol

## Abstract

Facial paralysis is a disabling condition with severe functional and aesthetic consequences. Facial paralysis affects approximately 1.8% of individuals over their lifetime, with approximately 30% of affected patients developing persistent deficits; among these, patients with permanent flaccid paralysis and severe facial asymmetry do not resolve with pharmacological treatment and require surgical intervention. It is specifically this surgically relevant subgroup that represents the target population of the SMILE framework. The SMILE framework (bypaSs of a facial nerve lesion through intra-body biocoMpatIbLE communication technologies) validates the feasibility of the communication infrastructure required to establish a functional neural bypass link between the healthy side and a surgically reinnervated contralateral side of the face. This work presents a preliminary interdisciplinary experimental and engineering approach underlying the SMILE framework, combining neurophysiological validation in 15 adult Wistar rats with the design of ultra-low-power intra-body communication links based on galvanic coupling (GC) and ultrasound (US). Microsurgical cuff electrodes were implanted around the buccal branch of the healthy facial nerve to record odor-evoked motor outputs. ENG signals recorded from the intact buccal branch of the facial nerve on one side were transmitted to the contralateral side, across the animal's facehead. Engineering evaluations demonstrated robust transmission capabilities, with the GC link achieving a mean equivalent SNR of 18.2 ± 0.6 dB and a mean normalized cross-correlation of *r* = 0.72 ± 0.09 between transmitted and reconstructed ENG signals, with MSE on the order of 10^−2^. The US link achieved an equivalent SNR around 12 dB with MSE around 5 · 10^−2^, supporting the feasibility of intra-body neural signal relay through biological tissues.

## 1 Introduction

Facial paralysis affects approximately 1.8% of individuals over their lifetime (Holland and Bernstein, 2014). Among these, approximately 30% of patients develop persistent and severe deficits, including severe facial asymmetry, that do not resolve with pharmacological treatment and ultimately require surgical intervention. Current surgical procedures for these flaccid cases, such as masseteric-facial neurorrhaphy, provide the required neural input but often fail to restore spontaneous emotional expression and resting symmetry (Biglioli, 2015a). The SMILE framework is specifically intended and designed for these patients, aiming to provide a minimally invasive alternative to achieve more physiologically faithful reanimation.

In those cases of unilateral facial palsy, whether idiopathic or resulting from nerve injury, that require surgery, surgical reinnervation to restore neural input to the facial muscles must be initiated before the onset of irreversible fibro-fatty degeneration of the mimetic musculature. This fibroadipose metaplasia typically becomes permanent if neural input is not restored within 12–18 months of onset, limiting the efficacy of subsequent reanimation procedures (Biglioli, 2015a). In the subset of patients with recent facial paralysis who do not recover spontaneously and require surgical intervention, current strategies rely on microsurgical procedures such as neurorrhaphies and nerve anastomoses to restore both voluntary and spontaneous movements, including smiling and eyelid closure. A common approach involves reinnervating the facial nerve trunk using donor nerves. For example, cross-face nerve grafts, using the sural nerve as a donor, can recruit branches from the contralateral facial nerve (Biglioli, 2015b). Alternatively, the masseteric nerve can be used to provide powerful innervation to the mimetic musculature, especially for smile restoration. In some complex reanimation protocols, masseteric-to-facial transfers are combined with cross-face nerve grafts approach to achieve both strength and spontaneity. While current microsurgical options, such as masseteric-to-facial neurorrhaphy, provide a crucial biological scaffold that preserves muscle tone and allows voluntary movement, they frequently fail to restore spontaneous emotional expression or natural resting symmetry (Park et al., 2020; Tarabbia et al., 2022; Biglioli, 2015a). Patients must consciously activate a donor nerve (e.g., by clenching the jaw) to initiate a smile, resulting in an expression that lacks subconscious emotional synchronization. The SMILE framework is designed to overcome this specific limitation as an adjuvant bio-electronic bypass. By continuously recording spontaneous physiological signals from the healthy hemi-face and transmitting them in real-time to modulate the newly reinnervated muscle targets, the system offers the necessary neural drive to achieve natural, symmetric, and emotionally synchronized mimetics.

Recent advances in intra-body communication technologies have opened new perspectives for minimally invasive functional nerve bypasses. Building upon the preliminary concept introduced in Vizziello et al. (2022), the SMILE framework (bypaSs of a facial nerve lesion through intra-body biocoMpatIbLE communication technologies) proposes a fully implantable system that records neural activity from the healthy facial nerve and transmits it to the contralateral injured side using body tissues as the propagation medium. By leveraging biocompatible transmission mechanisms, SMILE is designed to provide the foundational architecture for restoring symmetric facial muscle activation by validating a biocompatible transmission framework. In this framework, the present study validates the feasibility of a wireless neural signal acquisition and transmission system, which serves as the foundational structure for a closed-loop neuroprosthetic. While our current system demonstrates high-fidelity signal relay, the integration of a stimulation module to drive functional muscle reanimation remains a subsequent phase of this development.

The SMILE framework primarily focuses on extratemporal lesions of the facial nerve trunk, which represent a significant clinical challenge in unilateral facial paralysis. In these cases, the nerve is surgically accessible and direct neurorrhaphy or nerve grafting is technically feasible. Because the complex neuromuscular interactions, facial lesions cannot be fully investigated by *in vitro* models, mathematical simulations, or cell-based preparations alone, *in vivo* experimentation remains essential. In this study, the Wistar rat is adopted as an experimental model due to its anatomical and functional similarities to the human facial nerve system. Notably, in rats, the peripheral branches of the facial nerve emerge superficially above the muscle layers, allowing direct access to nerve terminals during surgical procedures (Pinto et al., 2022; Weinberg et al., 2016; Ali et al., 2020; Olmstead et al., 2015).

Salient olfactory inputs can modulate the central respiratory pattern generator, often inducing a shift toward active sniffing behavior. In rodents, this process is physiologically coupled to rhythmic whisking, a motor behavior driven by commands conveyed through the extratemporal branches of the facial nerve (Deschnes et al., 2012). Although whisking occurs independently of olfactory input, sensory stimuli, including odors, have been shown to significantly modulate whisking kinematics, increasing amplitude and curvature change in a time-locked manner (Renard et al., 2022). By exploiting this oro-facial circuit, the SMILE framework utilizes odor-evoked whisking as a functional readout. This allows for the assessment of efferent neural signals recorded directly from the peripheral facial nerve branches, providing a quantifiable measure of motor recovery following nerve repair.

Within this framework, the present study investigates the feasibility of the intra-body signal communication framework required for restoring facial function in a rat model of unilateral facial palsy by transmitting neural signals from the healthy hemi-face to the injured side. Neural activity is recorded from fine branches of the intact facial nerve using cuff electrodes designed for stable peripheral-nerve acquisition. After appropriate pre-processing, these signals are intended to be reproduced on the corresponding contralateral branches to drive symmetric muscle activation, thereby restoring bilateral whisking coordination and functional oro-facial responses to environmental stimuli.

To enable this neural bypass architecture, ultra-low-power intra-body communication technologies are considered. Conventional short-range radio frequency (RF) standards such as Bluetooth or Zigbee (Adibi, 2012) are generally unsuitable for intra-body networking due to high tissue attenuation and increased power requirements at GHz frequencies (Vizziello et al., 2024). As alternative modalities, galvanic coupling (GC) and ultrasonic (US) communications have emerged as suitable candidates for short-range implantable signal transmission. Coupling-based electromagnetic approaches typically operate below approximately 100 MHz and use μ*W*-level transmission power, improving safety and energy efficiency. GC technology can support propagation distances on the order of 20-30 cm with significantly lower energy consumption than RF transceivers (Vizziello et al., 2024). Ultrasonic communications represent a complementary modality, exploiting acoustic wave propagation in high-water-content biological tissues and enabling reliable intra-body transmission, with reported signal quality typically exceeding approximately 12 dB SNR under controlled experimental conditions (Vizziello et al., 2024, 2023).

**Figure 1:**
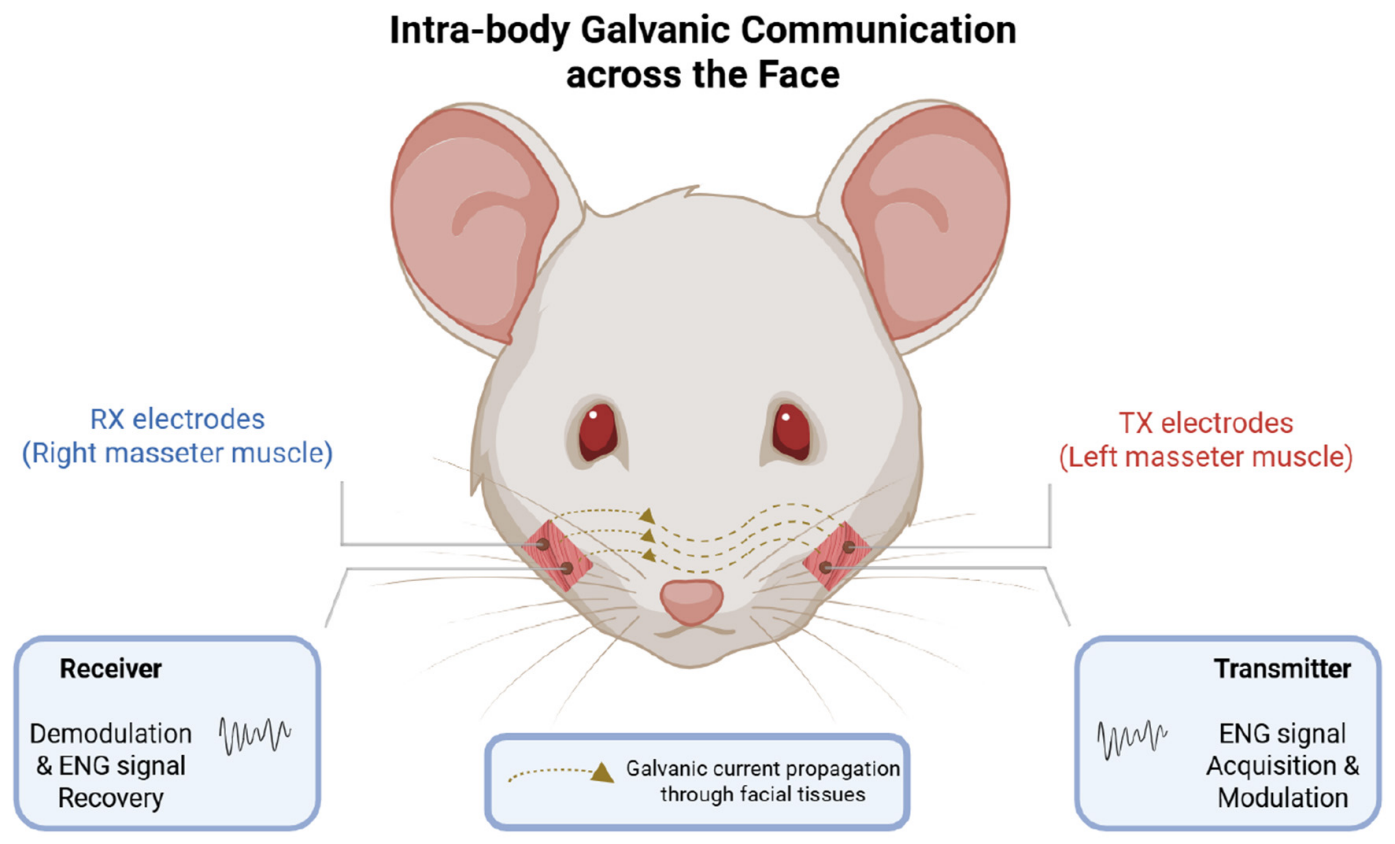
Physical principle of galvanic coupling (GC). Schematic representation of the communication mechanism to transmit neural signals through biological tissues between contralateral facial muscles. Created in BioRender.

In GC (Figure 1), low-intensity electrical currents in the 1 kHz-100 MHz range are injected into biological tissues through electrode pairs, generating a secondary current that propagates through the body and can be detected by distant receiver electrodes (Callejón et al., 2014; Seyedi et al., 2013). GC offers extremely low energy consumption, intrinsic immunity to external interference, and inherent data security, making it attractive for implantable systems (Vizziello et al., 2024).

Ultrasonic communications (UC) on the other hand, rely on the propagation of mechanical acoustic waves through tissues, which are well-suited for biological environments due to their high water content. Frequencies between 700 kHz and 5 MHz provide an effective trade-off between attenuation, directivity, and communication range (Santagati et al., 2015; Galluccio et al., 2017). Although ultrasonic propagation is subject to reflections and multipath effects, tissue absorption remains low and biologically safe, as evidenced by decades of clinical ultrasonography. Intra-body ultrasonic links have been demonstrated for both data transmission and energy harvesting in implantable systems (Seo et al., 2016), with recent studies highlighting the potential of diffusive propagation, multihop architectures, and vascular waveguides to extend communication range (Galluccio et al., 2018; Sciacca and Galluccio, 2020).

This paper presents the fundamental SMILE system architecture and focuses on the experimental validation of the biological protocol based on olfactory-driven whisking in rats with unilateral facial paralysis, together with the design considerations of biocompatible intra-body communication links.

## 2 Materials and methods

### 2.1 System overview and vibrissal experimental protocol

**Figure 2:**
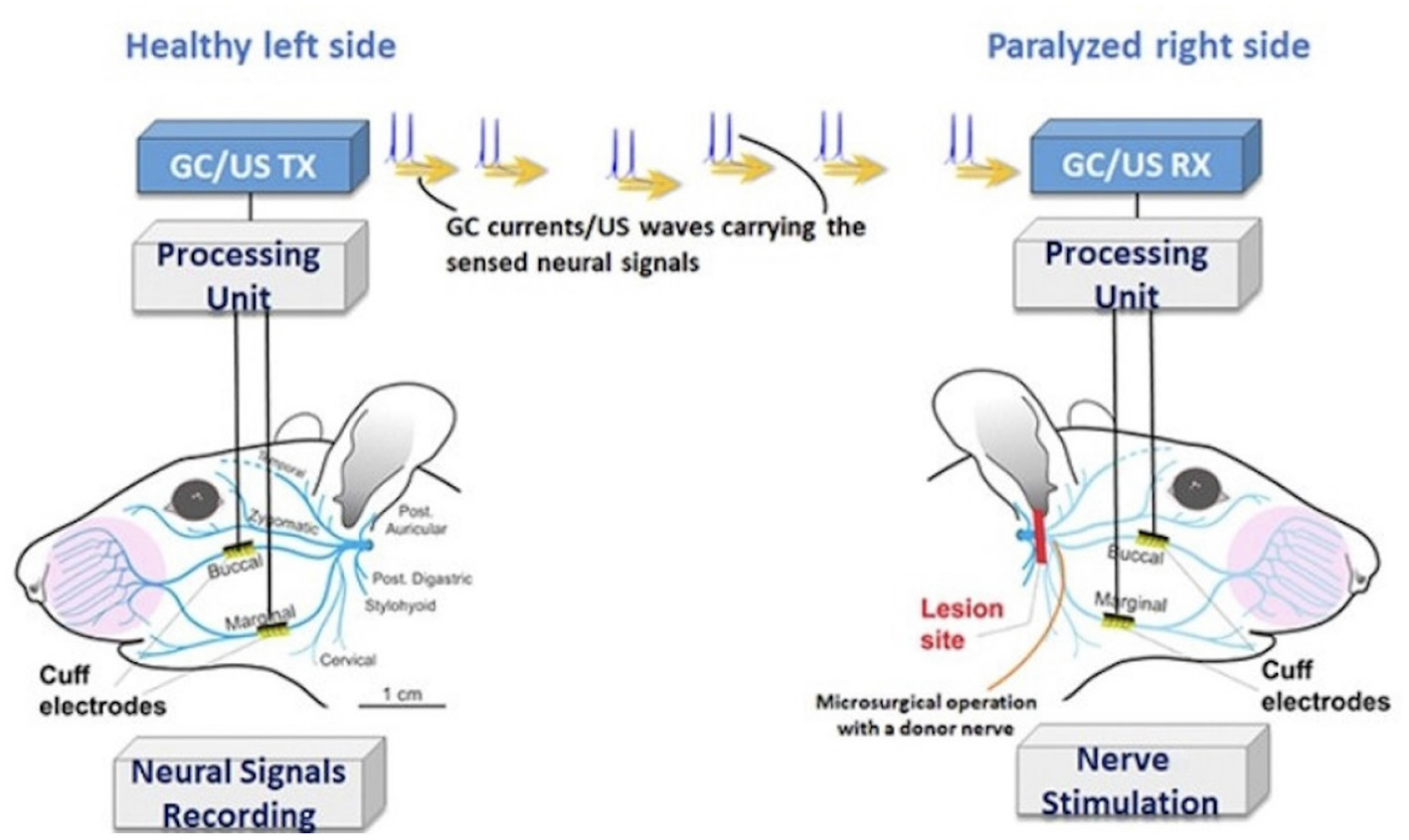
Schematic representation of the full SMILE framework, which is intended to restore facial symmetry via intra-body signal transmission from the healthy hemi-face **(left)** to the paralyzed side **(right)**. The figure illustrates the overall architecture of the project, including the conceptual recording, communication, and stimulation modules extending across the rat facial model from the healthy side to the contralateral lesion-related side. In the present study, we experimentally validated only the recording and transmission components on the left healthy side of the Wistar Han rat model, specifically the acute placement of cuff electrodes around the buccal branch of the intact facial nerve and the subsequent processing and transmission of the recorded ENG signals through biological tissues between contralateral facial muscles. The stimulation components on the right side of the animal model, as well as the facial nerve lesion-related elements, represent the intended future clinical architecture and were not implemented in this work.

The SMILE framework proposes a conceptual implantable neural communication system to restore facial nerve function by wirelessly relaying physiological signals from the healthy to the paralyzed side of the face. Only the recording and intra-body communication modules are realized and evaluated, while the contralateral stimulation components remain part of the intended system architecture. The architecture exploits the natural symmetry of facial movements, aiming to record neural signals from safe facial nerve branches, using minimally invasive multi-contact cuff electrodes, and forwarding this information across the face through ultra-low-power intra-body communication based on GC and/or US. On the pathological side, the perspective complete system requires a corresponding implant intended to decode relayed information, reconstruct stimulation patterns via a dedicated signal generation interface, and drive stimulating cuff electrodes placed on nerve branches distal to the lesion. Wallerian degeneration of the distal facial nerve branches must be prevented by performing an immediate end-to-end epineural neurorrhaphy between the masseteric nerve and the distal portion of the transected facial nerve trunk This procedure provides a ready reinnervation to the mimetic musculature, preventing irreversible muscle atrophy. This system-level design aims to reduce the number and invasiveness of conventional cross-face nerve grafting procedures, improve the control of fine, symmetric facial movements, and establish a flexible platform that can later integrate machine learning-based feature extraction, multihop GC/US links, and advanced power delivery schemes for broader applications to peripheral nerve lesions (Figure 2).

The SMILE framework is devised around three main pillars: (i) a Recording Module, (ii) a Processing and Communication Module, both of which are experimentally implemented and validated in this work, and (iii) an intended Stimulation Module, which is currently in the design phase and aimed at closing the neuroprosthetic loop in future experiments.

- **Recording module:** vibrissal whisking is a rhythmic high-frequency movement of the vibrissae primarily controlled by the buccal and marginal mandibular branches of the facial nerve, that can be experimentally elicited by using computer-controlled air valves to deliver scented air flows toward the rats' snout (Heaton et al., 2008, 2014; Hadlock et al., 2008). While whisking is a continuously performed motor behavior in rodents, the introduction of a salient odor stimulus significantly modulates the recruitment of nerve fibers within the buccal and marginal mandibular branches of the facial nerve. During this increased whisking response, the synchronized facial nerve activity of multiple nervous fibers is recorded as a compound action potential (CAP), which represents the summation of individual action potentials from the contributing fiber population. When recorded using cuff electrodes, this collective neural activity is measured as an electroneurographic (ENG) signal, whose waveform corresponds to the CAP generated by the recruited fibers. The recording module consists of multi-contact cuff electrodes chronically implanted around buccal and marginal branches of the healthy facial nerve to capture CAPs with minimal invasiveness and high spatial selectivity. These electrodes are interfaced with a low-noise, flexible analog front-end that provides amplification, filtering, and predistortion to compensate both the electrode-tissue transfer function and the weak, noisy nature of peripheral neural signals. Signals are sampled at appropriate rates (hundreds of Hertz to KHz), and their morphological, temporal, and spatial features are extracted.
- **Processing unit:** the processing unit performs lightweight conditioning of the recorded ENG signals to improve signal quality and adapt the data rate to the constraints of ultra-low-power intra-body communication. In the present implementation, signal processing is intentionally kept simple to minimize complexity and power consumption, in line with the preliminary nature of the experimental validation. Specifically, the acquired ENG signals are band-pass filtered within the characteristic ENG bandwidth (approximately 300 Hz–3 kHz) and processed by a notch filter to attenuate residual 50 Hz power-line interference. The conditioned signals are then downsampled to reduce the overall data rate, enabling efficient testing of GC and US transmission links while preserving the essential temporal and morphological features of the compound action potentials. This processing stage currently follows an amplify-and-forward paradigm, in which conditioned neural waveforms are directly prepared for transmission without higher-level interpretation.
- **Communication link:** the communication link bridges the healthy and paralyzed facial sides using ultra-low-power intra-body communication based on GC and US, which exploit the conductive and acoustic properties of biological tissues as propagation media. In this study, the communication link is implemented and experimentally validated for inter-side transmission of recorded ENG signals, without integration of the stimulation module. GC injects low-intensity currents in muscle or subcutaneous tissues through small biocompatible electrodes, while ultrasounds rely on miniaturized transducers operating in the sub-10 MHz range to balance attenuation, directionality, and coverage over distances of several centimeters. In this work, signal transmission is implemented using a single-hop, point-to-point link between one transmitter (TX) and one receiver (RX). Neural signals are uniformly quantized and modulated using a binary phase shift keying (BPSK) scheme, selected for its robustness and low power requirements, which are particularly suitable for intra-body communication scenarios. Basic synchronization and demodulation are employed to enable reliable reconstruction of the transmitted ENG signals at the receiver.
- **Stimulation module:** following the surgical approach described by Ali et al. (2020), a 4–5 mm incision will be made in the postauricular crease to perform. After adequate exposure, the main trunk of the facial nerve will be identified as it travels inferiorly from underneath the tendon of the digastric muscle tendon, at the point where it exits the stylomastoid foramen from the skull base. The stimulation module is envisioned to reconstruct, at the receiver side, neural stimulation patterns to be applied to the nerve branches distal to the lesion in order to restore symmetric whisking. The stimulation module is not implemented in the present study and is described only as part of the prospective system architecture required to close the loop in future work.
  In the proposed system architecture, a signal generation interface will be designed to translate the transmitted neural information into stimulation waveforms compatible with peripheral nerve activation. These waveforms will be delivered through cuff electrodes positioned on the buccal and marginal mandibular branches. In the future chronic model of facial palsy, these branches will be maintained via a concomitant masseteric-facial neurorrhaphy. This surgical procedure will serve as a biological scaffold to prevent Wallerian degeneration and muscle atrophy, while the SMILE system bypass provides the synchronized neural drive required for symmetric movement. By reproducing the temporal structure of motor compound activity observed on the healthy side, the stimulation module will aim to evoke coordinated muscular contractions that mirror physiological facial movements in the preclinical rat model. Its integration will be the primary objective of our future work and developments focused on stimulation safety, timing alignment, and adaptive parameter tuning to achieve effective and physiologically faithful facial reanimation.

### 2.2 Animal model and surgical procedures

All animal procedures were performed in accordance with the EU Directive 2010/63/EU and the Italian Legislative Decree 26/2014. The experimental protocol was approved by the Animal Welfare Body (OPBA) of the University of Pavia and authorized by the Italian Ministry of Health (Authorization n. 553.2024-PR). All efforts were made to minimize animal suffering and to reduce the number of animals used, in accordance with the 3Rs principles.

Before the experiment, rats were housed with food and water ad libitum, under a 12:12 h light/dark cycle. Neural data was collected from 15 adult Wistar Han rats (Charles River), aged 2–6 months, including both males and females. All surgical procedures were performed under aseptic conditions. Each rat was first anesthetized via inhalation of isoflurane (3%, for up to 90 s) and then maintained with an intraperitoneal administration of ketamine hydrochloride (90–100 mg/kg; Zootecnica Group S.p.a., Italy) and xilazine (20 mg/kg; Zootecnica Group S.p.a., Italy). Prior surgery, the animal received a single dose of analgesic (Meloxicam; 0,5mg/ml; Zootecnica Group S.p.a., Italy) to prevent pain and discomfort. At the end of the experiment, animals were euthanized in accordance with institutional and ministerial animal care protocols. Animal body temperature was maintained throughout the surgery and the entire experimental duration using a heating pad. During anesthesia, continuous respiratory rate and assessment of reflexes were performed, specifically considering the palpebral and pedal withdrawal reflexes.

**Figure 3:**
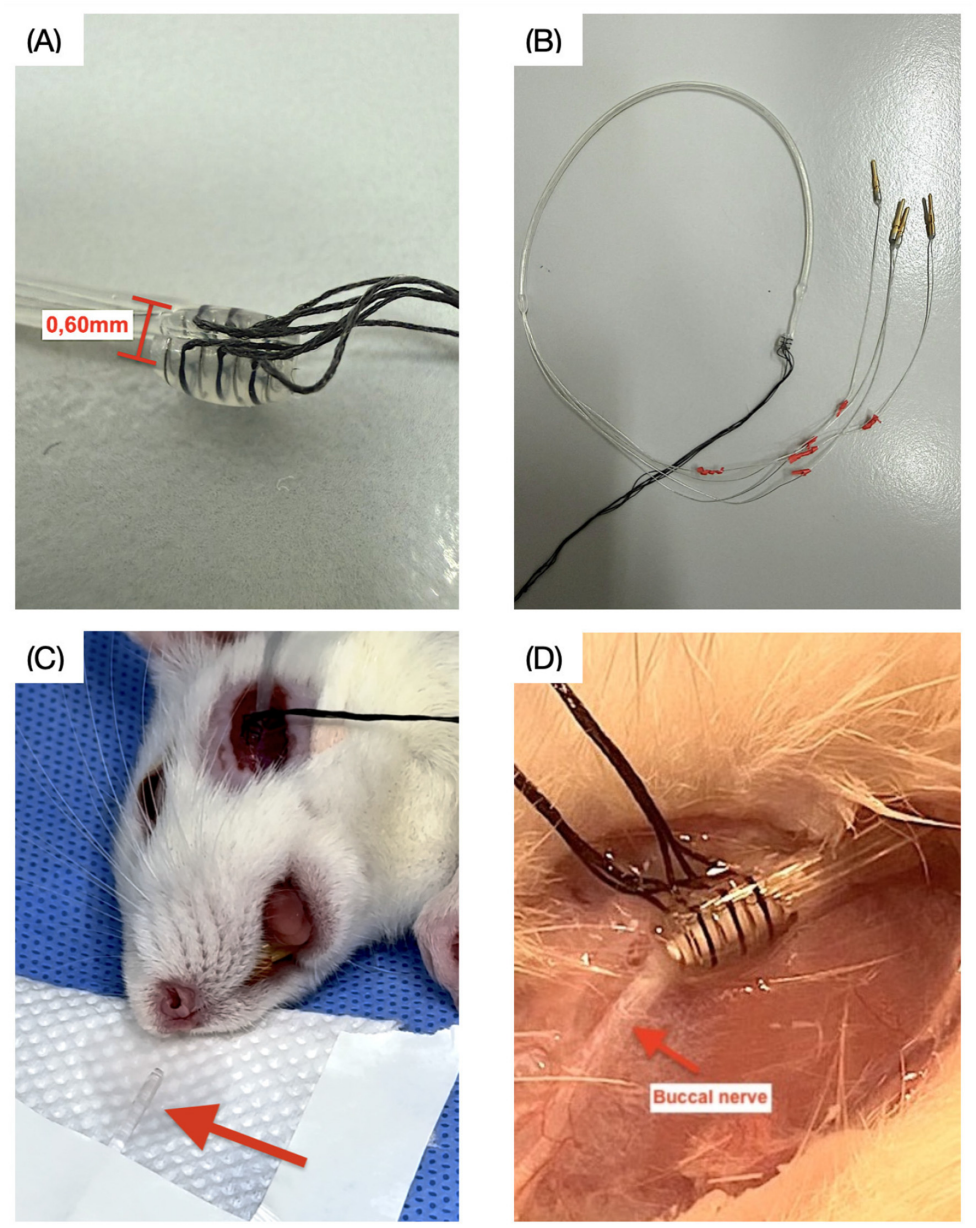
Custom cuff electrode design and surgical implantation. **(A)** Image of a cuff electrode (MicroProbes Life Science, Gaithersburg, MD) featuring a 0.60 mm inner diameter. **(B)** Detailed view of the cuff electrode assembly showing four electrode contacts, each soldered to a male gold pin. **(C)** *In vivo* surgical placement: the cuff electrode is implanted on the buccal nerve of the rat. The arrow indicates a micropipette positioned adjacent to the vibrissae (whiskers) for the delivery of ethanol droplets to elicit a whiskers response. **(D)** Surgical placement of the cuff electrode around the buccal branch of the rat facial nerve (red arrow). In all experiments, both facial nerves were left intact. The cuff electrode was used exclusively for ENG signals acquisition from the left buccal branch; no nerve stimulation was performed.

Once a surgical level of anesthesia was achieved, the target region on the left hemi-face, between the whisker pad and the ear, was carefully shaved and cleaned. The skin was disinfected, prior to incision, using a 70% antiseptic chlorhexidine solution (La Zootecnica Group S.p.a., Italy). Corneal hydration and eye infection prevention were ensured by applying a topical ophthalmic ointment (Lacrilube, Allergan, La Zootecnica Group S.p.a., Italy) to both eyes for the duration of the procedure. Subsequently, a small surgical incision of approximately 3 mm was made using a scalpel, just below the bony prominence of the left eye and extended toward the angle of the mandible (Placheta et al., 2015). The surgical area of interest was exposed by gently retracting the skin flaps. The buccal branch of the facial nerve in this region can be visualized beneath the skin. A cuff electrode (MicroProbes Life Science, Gaithersburg, MD) was gently positioned around the buccal branch using fine-tipped surgical forceps (Figure 3). To ensure stable electrode positioning and consistent nerve contact despite facial tissue movement, the silicone edges of the cuff were gently secured around the nerve using suture thread. This method promoted reliable signal acquisition while simultaneously preventing the risk of nerve constriction. Following irrigation of the area with sterile physiological saline (0.9%) and blotting it dry with sterile gauze, the animal was placed inside a Faraday cage.

### 2.3 Acquisition and processing of the signal

Facial nerve activity was recorded as neural CAPs propagating along the nerve surface. CAPs were acquired and recorded using cuff electrodes placed around the buccal branch of the facial nerve. Specifically, a cuff-electrode with four equally spaced concentric platinum contacts (100 μm each), measuring an inside diameter of 0.60 mm, was utilized for each rat. Three of the contacts were used as recording electrodes to monitor nerve activity, while one served as a reference electrode. Each contact was soldered to a male gold pin, which was connected via female gold pin to a 36-pin wire adapter (A79028-001, 2331, Omnetics Corp). An additional adapter's wire soldered to a male gold pin was used as the ground. The remaining cables from the adapter were enclosed within a braided sleeve for electrical insulation and mechanical protection. Before surgery, the cuff electrode was sterilized in 70% ethanol and then stored in sterile physiological saline (0.9%) until implantation. The adapter was connected to a RHD 32-Channel Recording headstage (RHD2132, Intan Technologies), which in turn was linked via a SPI cable to the acquisition board (Open Ephys), where the signals were digitized and recorded using GUI software (Open Ephys).

Nerve signals were induced by odor stimulation using 70% ethanol as a robust chemical trigger. This concentration was empirically selected, as lower concentrations and milder odorants failed to produce a reliable motor response in anesthetized animals. Ethanol was delivered 10 s after the start of the recording, through a tube connected distally to a borosilicate glass capillary tube (Hilgenberg GmbH, Malsfeld, Germany) and proximally to a syringe, which was manually operated by the experimenter. The movement of the whiskers on the left side of the animal was tracked using an infrared-illuminated camera specifically designed for monitoring rat behavior (Labeo Technologies Inc., Canada). The camera was positioned laterally to the left side of the animal, approximately 40 cm from the whisker pad, to ensure an optimal field of view. Videos were recorded at 226 fps, with a duration of approximately 15 s following stimulus delivery, and stored in .avi format. Video data were used for qualitative assessment of whisking behavior. The analog front-end included amplification, with a gain of 20, and band-pass filtering (0.3–3 kHz). The resulting data were then digitized at 30 kHz with 16-bit resolution using an Open Ephys Acquisition Board.

### 2.4 Intra-body communication design

#### 2.4.1 Galvanic coupling technology

GC was employed as a biocompatible intra-body communication method to relay ENG signals from the healthy to the paralyzed side of the face. GC relies on the injection of low-intensity electricg7 al currents through tissues, which act as the propagation medium. This technique enables ultra-low-power operation while maintaining minimal invasiveness, making it suitable for implantable neural interfaces. It should be noted that in this proof-of-concept study, GC transmission experiments were performed on deeply anesthetized or euthanized animal models. This approach allowed for the precise evaluation of signal propagation through biological tissue while eliminating potential artifacts or variability arising from spontaneous muscle contractions or tissue movement. The GC currents are extremely low, remaining well-below neural and muscular activation thresholds, and are therefore imperceptible and do not induce unintended stimulation of nearby tissues (Vizziello et al., 2024). Moreover, in line with previous studies, body motion does not significantly affect the GC channel frequency response (Vizziello et al., 2024).

**Electrodes and placement:** miniature biocompatible electrodes were used for both transmission and reception. Dupont male-to-male jumper wires were employed, in which the original metallic terminals were replaced with custom-made Alleima^^®^^ 316LVM contacts. The electrodes, with diameters of approximately 0.5 mm, were implanted in muscular tissue to exploit its high conductivity and ensure effective propagation of weak currents. Electrodes were placed with an inter-electrode distance of 1 cm within the masseter muscle on both the left and right sides of the face. To enable GC-based transmission of ENG signals from one side of the face to the other, TX electrodes were implanted within the left masseter muscle, while RX electrodes were implanted contralaterally within the right masseter muscle, with an inter-electrode distance of 1 cm at both sites.

**Frequency range and channel modeling:** GC operates by injecting low-intensity electrical currents into biological tissues, which act as a conductive propagation medium. In the proposed design, GC transmission is considered within the 1 kHz–100 MHz frequency range, which is widely adopted in intra-body communication to ensure compatibility with physiological signals while limiting interference with endogenous bioelectric activity. The anatomical structure of facial tissues, composed of layered skin, fat, muscle, and bone must be accounted for in the system design. For the rat facial model considered in this work, the inter-side propagation distance is on the order of a few centimeters, which is appropriate for short-range, ultra-low-power GC-based transmission. Channel characterization is informed by experimental measurements performed on *ex-vivo* and *in-vivo* tissues, as well as by established models reported in the literature (Vizziello et al., 2024). These characterizations guide the selection of electrode geometry, placement, transmission amplitude, and modulation bandwidth, enabling reliable conveyance of peripheral neural signals while maintaining minimal invasiveness and low power consumption.

**Signal transmission:** GC was employed to transmit the ENG recordings through intra-body links. ENG signals were initially acquired using cuff electrodes positioned around the buccal branch of the facial nerve. These neural signals were then transmitted via a GC-based testbed utilizing a sound card interface (Vizziello et al., 2020). A low-complexity BPSK modulation scheme was implemented, chosen for its low power requirements, which are essential for intra-body communication where energy efficiency is critical. Transmission experiments were carried out and the system demonstrated highly reliable performance, achieving near error-free signal transfer, as illustrated in Section 3.2.1. Single-link GC transmission between the healthy and paralyzed facial sides was tested and validated. The system successfully relayed neural activity across the targeted distance, maintaining a signal-to-noise ratio (SNR) well-above 10 dB, a value that ensures reliable transmission. These results indicate that GC can serve as a robust and low-power method for transmitting neural activity, highlighting its potential applicability in medical and implantable neural interface systems.

#### 2.4.2 Ultrasounds technology

US can be used for intra-body communication to transmit neural signals over medium/short distance. US signals propagate through biological tissues as mechanical waves, offering the possibility of directional transmission and simultaneous power delivery in miniaturized implants (Shah et al., 2025).

**Transducer technologies:** several types of ultrasonic transducers are available, including piezoelectric micromachined ultrasonic transducers (pMUTs) (Pappalardo et al., 2008; Birjis et al., 2022), capacitive micromachined ultrasonic transducers (cMUTs) (Pappalardo et al., 2008; Joseph et al., 2021), opto-ultrasonic transducers (Santagati and Melodia, 2014), and single-layer graphene (SLG) devices (Zhang et al., 2020). Each technology offers different trade-offs in terms of size, efficiency, and bandwidth, which are critical for miniaturized implantable applications.

**Frequency, directivity, and channel modeling:** the propagation of US waves in tissues is subject to attenuation, which increases with frequency, and to reflections at tissue boundaries, particularly critical in case of interfaces with bone. For intra-body applications over distances of a few centimeters, frequencies below 10 MHz are typically preferred to balance signal penetration, directivity, and transducer dimensions. Beam patterns can be engineered based on the ratio of radiating surface to wavelength to optimize coverage and focusing (Santagati et al., 2015). US propagation is affected by multipath components resulting from direct, reflected, and lateral waves. A preliminary characterization of the channel impulse response (CIR) in phantom propagation has been recently carried out (Sciacca and Galluccio, 2020).

**Modulation and energy considerations:** Techniques such as direct sequence spread spectrum (DSSS) and ultra-wideband (UWB) can be used to improve reliability and mitigate interference in multi-implant scenarios (Santagati et al., 2015). For miniaturized implants, simultaneous power and data transfer remains a key challenge. US enables energy delivery to millimeter-scale devices implanted several centimeters deep.

**Signal transmission:** US technology was used to transmit ENG through intra-body links. The setup utilized both Olympus V326-SU transducers operating at 3 MHz (Olympus Panametrics, 2026) and Mini-stack SM- PAK15222D2 (STEMINC, 2026), with each network node connected to a single transducer. Results were then averaged among these two technologies. Signal integrity was managed by a Mini-Circuits ZPUL-30P power amplifier for transmission and a ZFL-1000LN+ low-noise amplifier for reception. Data conversion was handled by an Ettus Research USRP N210—featuring high-speed 14-bit ADCs and 16-bit DACs—interfaced with a PC via GNU Radio. To prioritize energy efficiency for internal use, a low-complexity BPSK modulation scheme was employed. Experimental results confirmed high reliability, with the system achieving near error-free transmission and an SNR exceeding 15 dB.

## 3 Results

This section presents preliminary experimental results obtained to validate key components of the SMILE framework in a preclinical rat model. Specifically, we report (i) the acquisition of facial nerve ENG signals associated with odor-evoked whisking and (ii) the feasibility of transmitting such signals through ultra-low-power intra-body communication links based on GC and US.

**Figure 4:**
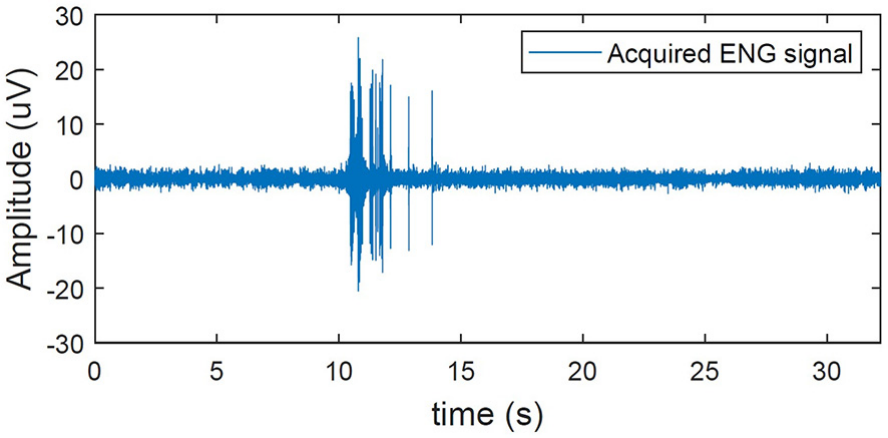
An example of a fraction of an acquired ENG signal recorded following odor stimulation delivered 10 s after the start of the recording, as described in Section 2.3.

Neural activity was recorded using cuff electrodes implanted on peripheral branches of the facial nerve, enabling the acquisition of motor Compound Action Potentials (mCAPs) associated with efferent motor commands. The recorded Electroneurogram (ENG) signals, as shown in Figure 4, provided a reliable measure of neural activity underlying whisking-related responses to sensory stimulation. Odor delivery using 70% ethanol consistently elicited a robust and stereotyped motor output, reflected by a clear increase in ENG signal amplitude compared to baseline conditions. This approach allowed for a distinct separation between resting and stimulated states and generated stable neural signals suitable for subsequent intra-body transmission experiments over biologically relevant distances.

### 3.1 ENG signal acquisition

ENG signals were acquired in different sessions, as described in Section 2.3, and then processed to enhance signal quality and identify neural activity. The digitized ENG signals were pre-processed and then imported into MATLAB (The Mathworks, Natick, USA). Since the frequency band of interest for an ENG signal is approximately between 300 Hz and 3 kHz, downsampling was applied to reduce the sampling frequency from 30 kHz to 6 kHz. Specifically, a decimation factor of 5 was applied, preceded by low-pass filtering designed to avoid aliasing and preserve the spectral integrity of the signal. Once the final sampling frequency of 6 kHz was reached, an infinite impulse response (IIR) notch filter was applied to attenuate traces of residual 50 Hz power-line interference.

**Figure 5:**
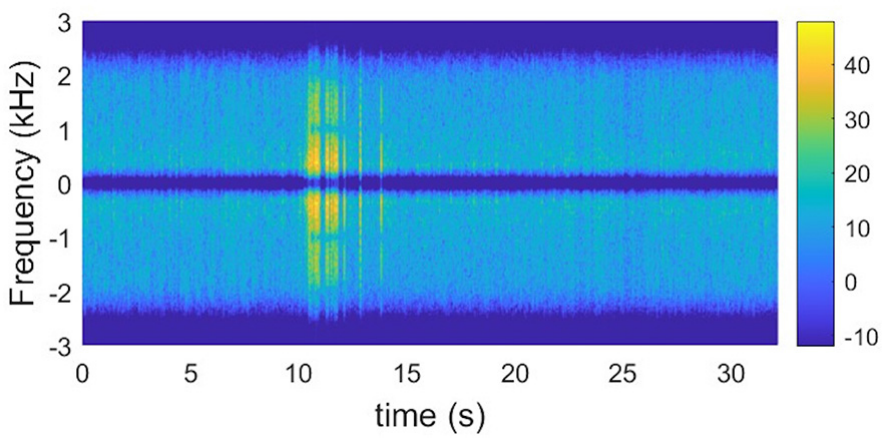
Short-Time Fourier Transform (STFT) of a representative Electroneurogram (ENG) signal. The plot illustrates the time-frequency evolution of motor Compound Action Potentials (mCAPs) during odor-evoked whisking. Amplitude values are expressed in dB with the color scale limited to 60 dB below the maximum.

An illustrative example of a part of an ENG signal acquired and processed according to the described procedure is shown in Figure 4. Inspection of the acquired time-domain trace reveals a distinct increase in neural activity concentrated between 10 and 15 s. To analyze the spectral dynamics of this signal over time and confirm the neural origin of the detected activity, the Short Time Fourier Transform (STFT) was applied. The result of this analysis is displayed in the corresponding spectrogram (Figure 5). To optimize visualization and interpretation, the amplitudes of the ENG signal were converted into decibels (dB). The color scale of the spectrogram was strategically limited to 60 dB below the maximum value. This choice emphasizes dominant neural components while reducing the impact of background noise. The spectrogram in Figure 5, precisely highlights the neural activity confined to the 10–15 s time window. Specifically, ENG activity is clearly visible within the expected bandwidth for nerve signals, which is [300, 2, 000] Hz. This agreement between time- and frequency-domain analyses validates the adopted processing pipeline and confirms its effectiveness in detecting odor-evoked ENG activity.

### 3.2 ENG signal transmission via intra-body communication

#### 3.2.1 GC transmission

A previously acquired ENG trace was transmitted through a GC intra-body communication link. The ENG signal was originally recorded over a duration of 30s at a sampling frequency of 30 kHz. For transmission purposes, a subset of 10,000 samples was extracted from the trace and downsampled, resulting in an effective sampling rate of 600 Hz.

**Figure 6:**
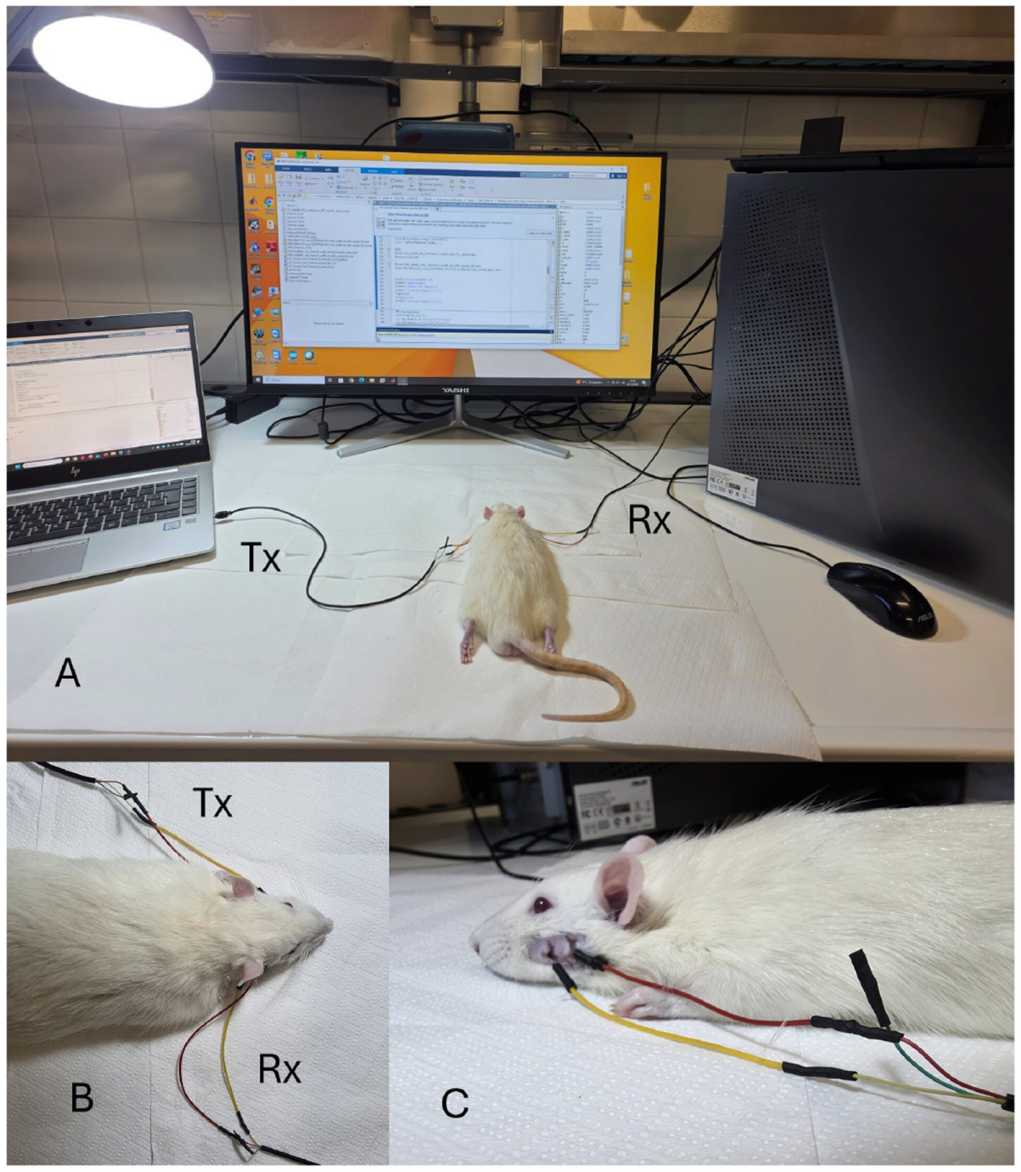
Experimental setup for galvanic coupling (GC) transmission. **(A)** General configuration of the TX and RX interfaces. **(B)** Superior and **(C)** lateral views of the rat specimen. Labels indicate the subcutaneous placement of the transmitting (TX) electrodes on the left side and receiving (RX) electrodes on the right side. On the right side, only electrode implantation was performed (no nerve transection) to validate signal transmission across the body structure.

Prior to transmission, the ENG samples were uniformly quantized using 8 bits per sample and modulated using BPSK. Transmission was carried out using standard miniaturized electrodes with a diameter of approximately 0.5 mm, as shown in Figure 6.

The GC transmission was evaluated on facial tissues (Figure 6), with an inter-electrode distance of 0.5 cm at both the transmitter and the receiver. The GC transmitted power *P*_*tx*_ was set to approximately 10 μW, within the low-power range typically adopted in intra-body coupling communication systems to support safe signal propagation (Vizziello et al., 2024). The transmission chain was implemented to ensure robust signal conveyance, following the transmission blocks detailed in (Vizziello et al., 2020). ENG signals were preprocessed using band-pass filtering within the characteristic peripheral nerve activity bandwidth and uniformly quantized prior to modulation. These include a preamble of 300 symbols for synchronization, pulse shaping and matched filtering using Square-Root Raised Cosine (SRRC) filters with a roll-off factor *r* = 0.2, and an oversampling factor of 16 for both the TX and RX. The received signal, based on experimental measurements, was down-converted to baseband using a normalized carrier frequency *f*_*c*_ = 0.3. The signal was then modulated in amplitude with a carrier of 10 KHz. Temporal synchronization was achieved via cross-correlation with the known preamble. The transmission parameters are summarized in Table 1. After de-quantization and fine alignment, system performance was evaluated over 10 independent runs. Results are averaged over 10 independent runs per animal, and 95% confidence intervals are computed using Student's T-distribution (Student, 1908).

**Table 1:** Parameters setting for GC transmission.

Parameter	Value
Carrier frequency *f*_*c*_ (KHz)	10
Waveform sampling frequency *f*_*s*_*a*__ (KHz)	48
Oversampling frequency *f*_*s*_ (no. of samples)	16
Sampling time *T*_*s*_ (ms)	0.66
RX oversampling frequency *f*_*s*_*rx*__ (no. of samples)	2
Roll-off of TRX filters *R*	0.2
Delay of TRX filters *D* (no. of samples)	8
QAM modulation order *M*	2
RX Wiener filter length *N*_*f*_ (no. of samples)	11
Modulated sequence *N* (no. of symbols)	10,000
Preamble length *N*_*pre*_ (no. of symbols)	1,000

The end-to-end latency of the complete transmission chain was estimated to be approximately 10 ms (Adame et al., 2021). This delay remains well-below both the intrinsic electromechanical delay of skeletal muscle contraction (10 s of milliseconds) and reported perceptual thresholds for detecting bilateral facial asynchrony (≥33 ms for blinking) (Kim et al., 2013), thereby supporting functional symmetry preservation.

The success criterion for the proposed transmission framework was the preservation fidelity between transmitted and reconstructed ENG signals, evaluated through bit error rate (BER), equivalent SNR, mean squared error (MSE), normalized cross-correlation, and qualitative waveform comparison. The stimulation module, while part of the overall SMILE system architecture, is not implemented in the present study and requires further dedicated design and experimental validation.

**Figure 7:**
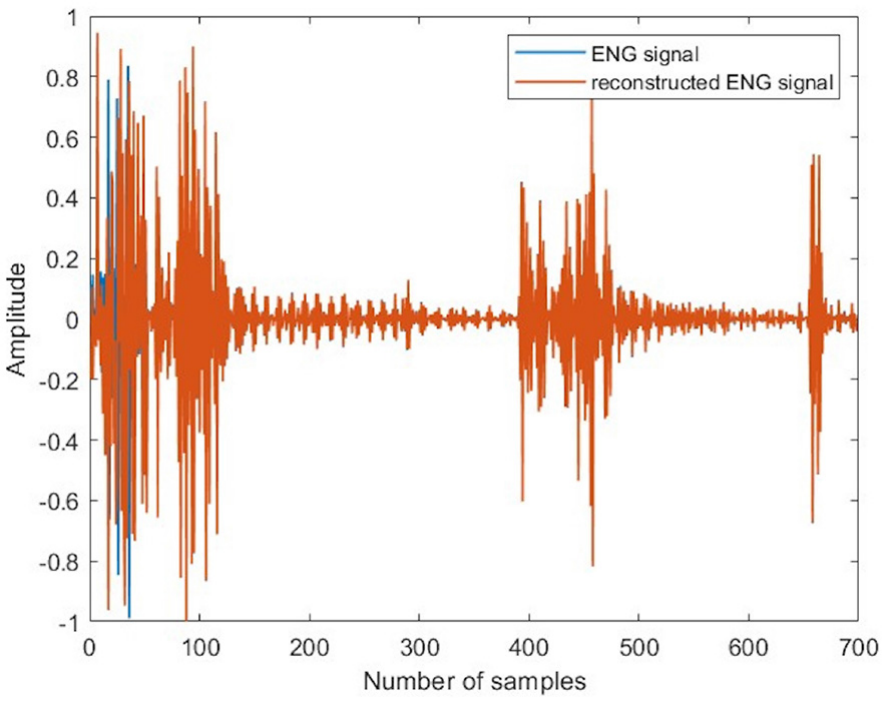
Representative comparison between original and GC-transmitted ENG signals. (Blue) Original ENG signal recorded from the healthy facial nerve and injected in the transmitter (TX) terminal; (Orange) corresponding signal received at the receiver (RX) site after transmission via Galvanic Coupling (GC). The alignment between the two traces demonstrates the high-fidelity reconstruction of neural firing patterns across the body volume.

Since nearly error-free transmission was observed, an extensive number of trials would be required to obtain statistically meaningful BER values. Therefore, performance was assessed in terms of the equivalent SNR, computed following the methodology described in Vizziello et al. (2020), which resulted in values on the order of 18 dB. The MSE between the transmitted ENG signal and the reconstructed received signal, evaluated at the bit level, was on the order of 10^−2^. Both the transmitted and reconstructed sequences were normalized in power prior to comparison. Figure 7 shows a qualitative comparison between the normalized original and reconstructed ENG signals. The measure of success was the correlation between the transmitted quantized whisker activation and the received signal.

For this representative trial, the maximum normalized cross-correlation was *r* = 0.7, and the signal-to-noise ratio was 18.41 dB, indicating high fidelity in both amplitude and waveform morphology. Across all trials, the mean SNR was 18.2 ± 0.6 dB, confirming consistent signal quality after transmission. Cross-correlation analysis across different trials shows a mean normalized correlation of *r* = 0.72 ± 0.09. These results indicate that the reconstructed signals preserve amplitude characteristics, and overall waveform structure across multiple trials. While some variability in correlation is observed, the majority of cases show strong structural similarity, supporting the robustness of the neural bypass. A slight mismatch can be observed at the beginning of the trace, while a substantial overlap between the two signals is achieved thereafter, confirming the robustness of the GC-based transmission.

#### 3.2.2 US transmission

**Figure 8:**
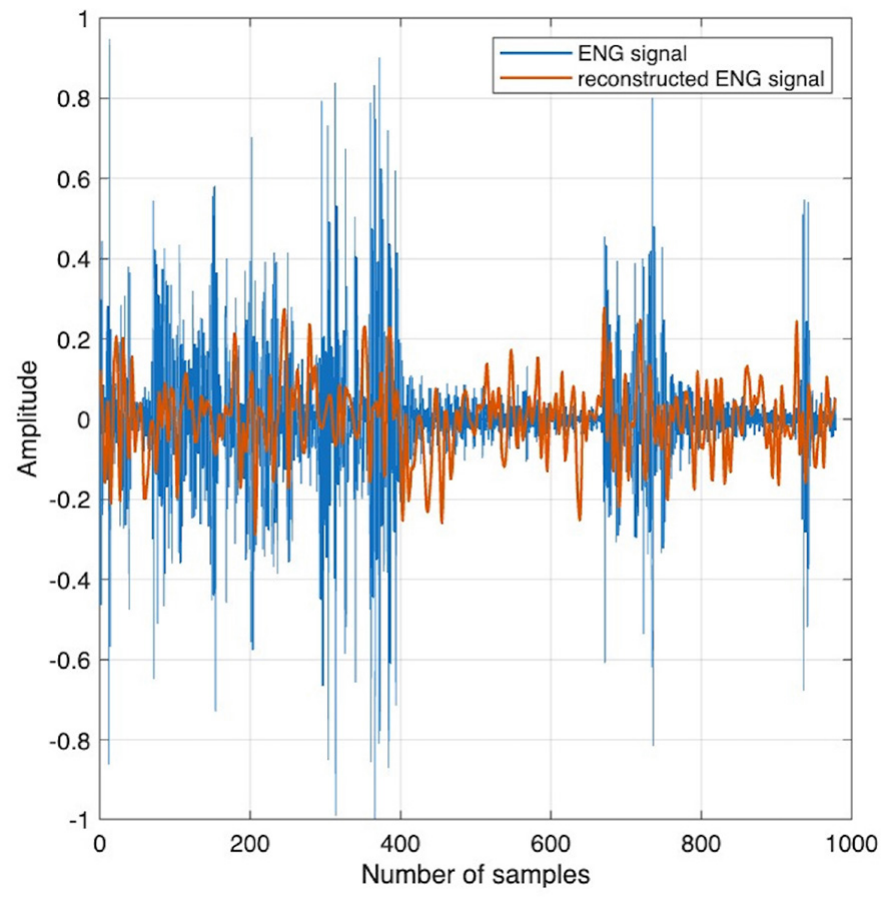
Characterization of the Ultrasonic (US) communication link. Representative comparison between the ENG signal and its reconstructed version after US transmission. The figure highlights the robustness of the mechanical wave propagation in the biological environment.

The ENG trace was transmitted using an US intra-body communication link. US transmission on the rat model was not feasible due to large transducer size, so a tissue-mimicking ballistic gel phantom with embedded bone structure (Sciacca and Galluccio, 2020) was used. Although this simplified setting is presented as a proof-of-concept about use of US technology in nerve lesion recovery, it establishes a solid foundation for use of intra-body US communications across biological tissues; ongoing advances in highly miniaturized transducers, such as cMUT (Pappalardo et al., 2008; Joseph et al., 2021) and pMUT (Pappalardo et al., 2008; Birjis et al., 2022), are expected to enable future investigations that further capture the complexity and variability of *in vivo* conditions relevant to implantable applications. For comparison with GC, the same general transmission framework as used for GC was adopted, including BPSK modulation and basic synchronization. The ENG signal was pre-processed, normalized, and uniformly quantized using six bits per sample before modulation. The signal was then modulated in amplitude with a carrier of 3 MHz. After de-quantization and fine alignment, system performance was evaluated. The equivalent SNR was found to be approximately 12.63 dB, with a corresponding MSE in the order of 5.46·10^−2^. To further enhance the signal quality, a Wavelet-based denoising stage (using the db4 mother wavelet at two levels) was applied to the reconstructed sequence. As shown in Figure 8, a qualitative comparison between the original and reconstructed ENG signals demonstrates a trend overlap. Despite the modulation and the real constrained measured channel, the system successfully preserved the morphological features of the ENG trace. However, a significant attenuation as compared to the GC case is observed.

## 4 Conclusions

### 4.1 Key findings

This study demonstrated the feasibility of recording odor-evoked ENG signals from the intact facial nerve of rats and transmitting them across the body to the contralateral side using biocompatible intra-body communication links based on GC and US. ENG acquisition using cuff electrodes provided a stable and high-fidelity readout of neural motor activity. GC transmission enabled reliable, near error-free signal propagation over short intra-face distances, with a high equivalent SNR. US transmission also preserved the morphological features of the ENG signals, albeit exhibiting higher attenuation as compared to GC. Although moderate variability in correlation values was observed, the majority of trials demonstrate strong structural similarity, supporting the robustness of the proposed framework. These results confirm that physiological neural activity recorded from an intact peripheral nerve can be reliably relayed through living tissues to the contralateral side using ultra-low-power communication techniques, establishing the foundational signal backbone for future implementation in a model of unilateral facial palsy.

### 4.2 Context and novelty

Previous studies have explored intra-body communication primarily with synthetic or simplified bio-signals. This work advances the field by demonstrating the relay of real ENG associated with functional facial movements, providing a direct proof-of-concept for a minimally invasive neural bypass. The novelty lies in combining neurophysiological validation with engineering design to implement low-power, biocompatible communication links suitable for implantable systems, an approach not previously reported in the context of facial nerve reanimation.

### 4.3 Limitations

The present study is limited by the small sample size and the use of a preclinical rat model, which, although anatomically relevant, does not fully replicate human facial nerve architecture. In addition, transmission experiments were conducted over short distances within a single-hop configuration. The system does not yet include a stimulation module to reproduce neural activity on the lesioned side, and long-term biocompatibility and stability of the implanted devices were not evaluated. While the current proof-of-concept utilizes external interfaces for data acquisition and monitoring, the communication link itself remains intra-body, as signal propagation occurs through the heterogeneous biological tissue volume via galvanic coupling or ultrasonic pathways. The current work focuses on the acute validation of the wireless signal architecture, the challenges associated with chronic electrode implantation must be considered. During our preliminary phase, we evaluated multiple cuff diameters (including 0.75 mm) and different geometric arrangements (2- and 4-electrode configurations) to optimize the balance between signal-to-noise ratio (SNR) and the risk of nerve compression. Our findings indicated that a 0.6 mm inner diameter provided the most secure interface for the buccal branch of the rat facial nerve, maintaining sufficient clearance to prevent constriction while ensuring signal quality. Importantly, the choice of cuff electrodes as our interface reliable device is supported by a growing body of literature demonstrating their efficacy as stable, long-term neural interfaces. Specifically, clinical and experimental evidence confirms that cuff electrodes can maintain signal integrity and spatial resolution over extended periods, while remaining biocompatible with peripheral nerves (Rijnbeek et al., 2018; Christie and de Leeuw, 2017). It must be noted that all *in vivo* validations in the present study were conducted under acute experimental conditions. While this was sufficient to prove the immediate technical feasibility of the recording and communication modules, the chronic long-term effects of the cuff electrode implantation, such as mechanical nerve compression, fibrotic encapsulation, or chronic signal degradation, were not evaluated. Future development phases of the SMILE framework will transition to chronic survival models to rigorously evaluate long-term biocompatibility, device stability, and functional outcomes over extended periods. Despite these limitations, the study demonstrates the feasibility of reliable neural signal acquisition and transmission through intra-body communication, establishing a foundational signal backbone for future functional facial restoration. Future work will focus on the development and integration of a stimulation interface, including precise timing alignment and adaptive parameter tuning, to achieve physiologically accurate and symmetric facial movements.

### 4.4 Future directions

The SMILE framework envisioned a system for acquiring and faithfully transmitting neural signals to the contralateral hemiface. While the current stage of development does not yet include a stimulation module to restore vibrissae movement on the injured hemiface in response to odorant stimuli, this study validates the framework's core architecture using a rat model. Our first effort will therefore be in the implementation of the stimulation module. While our current study validates the SMILE framework using the rat model, we acknowledge the anatomical differences intrinsic in human subjects, specifically regarding the increased depth of the facial nerve and the complexity of surrounding soft tissues. The surgical access to the facial nerve in humans differs from the superficial peripheral branches utilized in rodents; however, the use of nerve-cuff interfaces is clinically proven for peripheral nerve applications (Abiri et al., 2022). Anatomical studies in feline models, which closely mirror human facial nerve organization and fiber orientation (Gacek and Radpour, 1982; Dresner et al., 2006), support the technical feasibility of applying cuff electrodes to restore facial function in larger, more complex structures. Future translational efforts will focus on optimizing surgical access and electrode dimensions to adapt this communication architecture for the clinical requirements of human facial nerve rehabilitation.

Future research will also focus on integrating multi-link or hybrid GC/US networks to optimize reliability and energy efficiency, extending the approach to larger animal models, and incorporating the stimulation module to restore functional whisking. Advanced signal processing, including feature extraction and machine learning, may further enhance signal compression, decoding accuracy, and stimulation fidelity. Future work will focus on the design and integration of stimulation modules toward a fully closed-loop neural bypass architecture. Furthermore, we will investigate in clinical tests electric field distribution in facial tissue and evaluate risks of unintended neural activation, rectification effects, or tissue heating. Ultimately, this framework demonstrates the feasibility of the proposed approach for acute neural interfacing experiments, and shows promise for future neural interfacing applications, although chronic validation remains necessary.

In conclusion, while the current work does not yet constitute a fully integrated closed-loop reanimation system, it successfully validates the acquisition and intra-body transmission of neural signals required for such a system. By demonstrating high-fidelity transmission of ENG signals from the intact facial nerve of one side to the other side through biological tissues, this work provides the essential proof-of-concept toward the future implementation of the complete SMILE framework for functional facial nerve rehabilitation.

## Data Availability

The raw data supporting the conclusions of this article will be made available by the authors, without undue reservation.
